# Exploring Low‐Intensity Focused‐ultrasound Neuromodulation in Alzheimer's Disease

**DOI:** 10.1002/alz70858_100580

**Published:** 2025-12-25

**Authors:** Ali R Rezai, Camila Vieira Ligo Teixeira, Manish Ranjan, Geoffrey Adams, Tasneem Arsiwala, Victor Finomore, Marc W Haut

**Affiliations:** ^1^ Rockefeller Neuroscience Institute, West Virginia University, Morgantown, WV, USA

## Abstract

**Background:**

Alzheimer's disease (AD) presents a major public health challenge with few effective therapeutic options. Monoclonal antibodies targeting β‐amyloid show promise, but their clinical efficacy remains modest. As such, combining pharmacological and non‐pharmacological interventions to slow cognitive decline has become a key area of research. Low‐intensity focused ultrasound (LIFU), a non‐invasive neuromodulation technique with excellent spatio‐temporal precision, has emerged as a potential therapeutic option for AD. Our group has had success in using LIFU for BBBOpening This pilot study aims to assess the safety, feasibility, and tolerability of LIFU as an adjunct treatment for individuals with mild cognitive impairment (MCI) due to AD (NCT05997030).

**Methods:**

This open‐label pilot study includes participants with amyloid positive MCI who undergo a single session of LIFU targeting the nucleus accumbens, hippocampus, and entorhinal cortex. Safety is assessed using cognitive evaluations, brain MRI, and FDG PET scans at baseline and follow‐up intervals to monitor any adverse events and metabolic and cognitive changes.

**Results:**

Three amyloid positive patients with MCI (two males and one female) underwent a single LIFU neuromodulation session. The FUS procedure was safe and well‐tolerated. There were no new neurological or imaging abnormalities. Cognitive assessments showed no changes in memory and attention, but there were slight increases in scores at the short‐term follow‐up period of 07 and 30 days after the FUS procedure. The results will be updated as the clinical trial progresses and presented at the meeting.

**Conclusions:**

These preliminary findings suggest that LIFU neuromodulation is safe and may offer cognitive benefits for individuals with amyloid‐positive MCI. This study highlights the promise of FUS technology as a novel adjuvant therapeutic approach in AD.

